# Aperiodic neural activity distinguishes between phasic and tonic REM sleep

**DOI:** 10.1111/jsr.14439

**Published:** 2024-12-26

**Authors:** Yevgenia Rosenblum, Tamás Bogdány, Lili Benedikta Nádasy, Xinyuan Chen, Ilona Kovács, Ferenc Gombos, Péter Ujma, Róbert Bódizs, Nico Adelhöfer, Péter Simor, Martin Dresler

**Affiliations:** ^1^ Radboud University Medical Centre, Donders Institute for Brain, Cognition and Behavior Nijmegen Netherlands; ^2^ Institute of Psychology, ELTE Eötvös Loránd University Budapest Hungary; ^3^ Doctoral School of Psychology, ELTE, Eötvös Loránd University Budapest Hungary; ^4^ HUN‐REN‐ELTE‐PPKE Adolescent Development Research Group Faculty of Education and Psychology, Eötvös Loránd University Budapest Hungary; ^5^ Pázmány Péter Catholic University, Department of General Psychology Budapest Hungary; ^6^ Semmelweis University, Institute of Behavioural Sciences Budapest Hungary

**Keywords:** 1/f, aperiodic activity, electroencephalogram, polysomnography, rapid eye movement, rapid eye movement sleep, scale‐free, sleep

## Abstract

Traditionally categorized as a uniform sleep phase, rapid eye movement sleep exhibits substantial heterogeneity with its phasic and tonic constituents showing marked differences regarding many characteristics. Here, we investigate how tonic and phasic states differ with respect to aperiodic neural activity, a marker of arousal and sleep. Rapid eye movement sleep heterogeneity was assessed using either binary phasic‐tonic (*n* = 97) or continuous (in 60/97 participants) approach. Slopes of the aperiodic power component were measured in the low (2–30 Hz, *n* = 97) and high (30–48 Hz, *n* = 60/97) frequency bands with the Irregularly Resampled Auto‐Spectral Analysis applied on electroencephalography. Rapid eye movement amplitudes were quantified with the YASA applied on electrooculography (*n* = 60/97). The binary approach revealed that the phasic state is characterized by steeper low‐band slopes with small effect sizes and some topographical heterogeneity over datasets. High‐band aperiodic slopes were flatter in the phasic versus tonic state with medium‐to‐large effect sizes over all areas in both datasets. The continuous approach confirmed these findings. The temporal analysis within rapid eye movement episodes revealed that aperiodic activity preceding or following EM events did not cross‐correlate with eye movement amplitudes. This study demonstrates that aperiodic slopes can serve as a reliable marker able to differentiate between phasic and tonic constituents of rapid eye movement sleep and reflect phasic rapid eye movement event intensity. However, rapid eye movement events could not be predicted by preceding aperiodic activity and vice versa, at least not with scalp electroencephalography.

## Graphical abstract

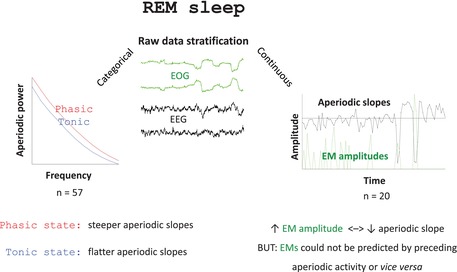

## 1 INTRODUCTION

### 1.1 Rapid eye movement (REM) sleep states

Nocturnal human sleep has a cycling nature where each cycle comprises an episode of non‐rapid eye movement (NREM) sleep followed by an episode of REM sleep (Aserinsky & Kleitman, 1953; Dement & Kleitman, 1957). REM sleep in turn cycles between phasic states characterized by bursts of eye movements (EMs) and tonic states that consist of longer and more quiescent segments (Ermis et al., 2010; Simor et al., 2020). Phasic and tonic periods exhibit remarkable differences concerning mental experience, environmental alertness, spontaneous and evoked cortical activity, information processing, neuronal network and autonomic (e.g. heart rate, skin conductance, respiration) activity (Simor et al., 2018, 2020). For example, phasic states show internally driven sensorimotor processing detached from the surroundings, and therefore are often tagged as “offline” periods (Datta et al., 2013). Tonic states are more responsive to external stimuli as evidenced by awakening and arousal thresholds, and thus are often tagged as “online” periods (Simor et al., 2020). Notably, rather than discrete states, phasic and tonic states are sometimes seen as “ends of a continuum” (Bueno‐Junior et al., 2023). In this study, we investigate whether heterogeneity of REM sleep (defined in either discrete or non‐discrete ways) is reflected by aperiodic neural activity, a marker of states of vigilance, sleep stages, sleep depth and sleep intensity.

### 1.2 Aperiodic activity

Aperiodic neural activity is a distinct type of brain dynamic that reflects the overall frequency composition of the neural signal, which is not dominated by any specific frequency (Bódizs et al., 2024). Aperiodic dynamics follow a power‐law function, where power decreases with increasing frequency (He, 2014). The steepness of this decay is approximated by the spectral exponent, which is equivalent to the slope of the spectrum when plotted in the log–log space (Gerster et al., 2022). Steeper (more negative) slopes indicate that the spectral power is relatively stronger in slower frequencies and relatively weaker in faster ones and vice versa (Bódizs et al., 2024).

In 2017, Gao et al. suggested that high‐band (30–50 Hz) aperiodic slopes reflect the balance between excitatory and inhibitory neural currents, which in turn defines a specific arousal state and a conscious experience of an organism. Following this report, aperiodic activity has received increased attention, and now is being seen as a window into diverse neural processes associated with cognitive tasks, age and disease (Bódizs et al., 2021; Höhn et al., 2024; Voytek & Knight, 2015). In the field of sleep research, it has been shown that aperiodic slopes differentiate between sleep stages such that high‐band slopes are steeper during REM than NREM sleep, whereas low‐band and broadband slopes show the opposite pattern (Lendner et al., 2020; Kozhemiako et al., 2022; Miskovic et al., 2019; Schneider et al., 2022). Given the ability of aperiodic activity to reflect many parameters of sleep, such as intensity, depth, stages and cycles of sleep (for review, see Bódizs et al., 2024), we hypothesize that it would further differentiate between REM sleep constituents.

### 1.3 Aims and hypotheses

To assess whether REM sleep heterogeneity is reflected by aperiodic activity, we used both binary tonic‐phasic categorization and the continuous approach that quantified EM amplitudes. We hypothesized that the phasic state/higher EM amplitudes would be associated with steeper aperiodic slopes in the low‐frequency band. This is based on the previous finding on increased delta oscillations in phasic versus tonic states (Simor et al., 2020), as such a difference implies a relative predominance of lower frequencies in phasic spectral power and, thus, a steeper decay. Regarding the high‐frequency band, we hypothesized that the phasic states would be linked to flatter aperiodic slopes based on a reported increase in gamma oscillations in phasic versus tonic states (Simor et al., 2020). Such a difference implies the relative predominance of higher frequencies in this band and, thus, slower/flatter spectral decay. In addition, we assessed temporal relationships between oculomotor and aperiodic activities across a duration of single REM sleep episodes. Here, we used cross‐correlations, assuming that its shape would reveal information concerning the possible direction (leading versus lagging) of the influence between the two time series.

## 2 METHODS

### 2.1 Datasets

The REM sleep heterogeneity was assessed using either a binary phasic‐tonic (*n* = 97) or continuous approach (*n* = 60/97). For the binary categorization, we first used three independently collected open‐source datasets of epoched polysomnographic recordings of healthy young individuals overall comprising 57 participants aged 21.5 ± 1.4 years. These datasets are described in Simor et al. (2018, 2019, 2021), and here are referred to as Datasets 1–3. Next, we replicated all the analyses using a non‐open‐source dataset of 40 healthy participants of a broader age range (31.5 ± 10.25 years). This dataset is described in Rosenblum, Bovy et al. (2023) as the control group, and here is referred to as Dataset 4. For the continuous approach, we used Datasets 3 and 4. Information about the studies, participants, polysomnographic devices as well as the links to the publicly available data are reported in Table 1. All studies were approved by the corresponding Ethics committee. All participants gave written informed consent.

**TABLE 1:** Study and device information for each dataset.

Characteristic	Dataset 1	Dataset 2	Dataset 3	Dataset 4
Described in:	Simor et al. (2018), Simor et al. (2021)	van der Wijk et al. (2020), Simor et al. (2021)	Simor et al. (2019)	Rosenblum, Bovy, et al. (2023) (as the control group)
Epoched data availability:	Open access on https://osf.io/2vptx		Open access on https://osf.io/9k5hb	Available for authors but non‐open access
Continuous data availability:	Non‐available for authors		Available for authors but non open‐access	
Approved by	The Ethical Committee of the Semmelweis University, Hungary	The United Ethical Review Committee for Research in Psychology, Hungary (EBKEB 2016/077)	The Ethical Committee of the Pázmány Péter Catholic University for Psychological Experiments. Hungary	The Ethics Committee of the University of Munich, Germany
No. participants	20	17	20	40
Age, years	21.72 ± 1.36	21.46 ± 1.43	21.19 ± 0.43	31.5 ± 10.25
Gender, female, %	50	70	44	53
Polysomnographic device	Brain‐Quick BQ 132S (Micromed, Mogliano Veneto, Italy)	Micromed SD LTM 32 Bs (Micromed S.p.A., Mogliano Veneto, Italy)	BQ KIT SD, LTM 128 EXPRESS (2 × 64 channels in master and slave mode; Micromed, Mogliano Veneto, Italy)	JE‐209A amplifier (Nihon Kohden, Tokyo, Japan), with 128Ch‐BrainCap (EasyCap GmbH, Herrsching, Germany)
No. channels	19	17	128	128
Referenced to	Mathematically linked mastoids (A1, A2)		Fronto‐centrally placed electrode	The average of all electrodes
Sample rate, Hz	1024	512	512	200

The participants slept wearing a polysomnographic device in a sleep laboratory with an adaptation night before the examination night. Only the second‐night data were analysed. Sleep was scored by independent experts according to the AASM standards (Iber, 2007). Epochs with electromyogram (EMG) and electroencephalogram (EEG) artefacts were manually excluded by an experienced scorer before all automatic analyses, so that only clean REM epochs were used for further analysis.

### 2.2 REM sleep binary segmentation

We used EEG data preprocessed as described elsewhere (Simor et al., 2018, 2019, 2021; van der Wijk et al., 2020). Briefly, first, the Independent Component Analysis (ICA) was applied to the data to correct for oculomotor artefacts (Simor et al., 2021). Then, the data were semi‐manually inspected for rapid EMs with a custom‐made software tool for full‐night sleep EEG analysis (FerciosEEGPlus, Ferenc Gombos 2008–2017). EMs were visually identified in non‐overlapping 4‐s time windows based on the presence of electrooculogram (EOG) deflections of amplitude above 150 μV and shorter than 500 ms. A 4‐s‐long segment was categorized as phasic if at least two consecutive EMs were detected in adjacent 2‐s time windows. Segments were scored as tonic when no EMs occurred (EOG deflections below 25 μV) in adjacent 2‐s time windows. To avoid contamination between the two microstates, segments were only selected if they were at least 8 s apart from each other.

### 2.3 Spectral power

Offline EEG data analyses were carried out with MATLAB (version R2021b, The MathWorks, Natick, MA, USA), using the Fieldtrip toolbox (Oostenveld et al., 2011) and custom‐made scripts. For each REM state of each participant, we averaged the preprocessed EEG signal over four topographical areas: frontal (Fz, F3, F4); central (Cz, C3, C4); parietal (Pz, P3, P4); and occipital (O1, O2) separately to reduce the number of statistical comparisons.

We calculated the total spectral power of every 4‐s EEG segment and differentiated it into its aperiodic (i.e. fractal) and oscillatory components using the Irregularly Resampled Auto‐Spectral Analysis (IRASA; Wen & Liu, 2016), the approach embedded in the Fieldtrip toolbox. To implement the algorithm, we used the *ft_freqanalysis* function of the Fieldtrip toolbox as described elsewhere (Rosenblum et al., 2024). Then, the aperiodic power component was transformed to log–log coordinates and its slope was calculated to estimate the power‐law exponent (the rate of spectral decay), using the function *logfit* (Lansey, 2020; available on https://osf.io/zhyf7).

The signal was analysed in the low‐ and high‐frequency bands separately. The low‐band was defined as 2–30 Hz, being a representative of the typical sleep frequency range (0.3–30 Hz), with the exclusion of frequencies < 2 Hz in order to control for the possibility that slow frequency activity during phasic periods is contaminated by EMs whose potentials mainly extend over 0.3–2 Hz (Tan et al., 2001) while keeping slow‐frequency activity that is different from EMs, namely, slow‐frequency, delta and delta‐theta (sawtooth waves) activity inherent to REM sleep (Bernardi et al., 2019). Given that EMs can affect the signal even after the ICA, we report the results for the signal filtered in the 5–30‐Hz band (which are comparable to those obtained for the 2–30‐Hz band, Figure S1).

The high‐band (30–48 Hz) was analysed as this range has been used for reliable discrimination between wakefulness and REM sleep (Lendner et al., 2020). Likewise, the 30–50‐Hz band was previously proposed to indicate excitation‐to‐inhibition balance (Gao et al., 2017). The high‐band analysis was performed for Datasets 3–4 only as the open access data for Datasets 1 and 2 had been filtered in the 0.5–35‐Hz range.

**FIGURE 1:**
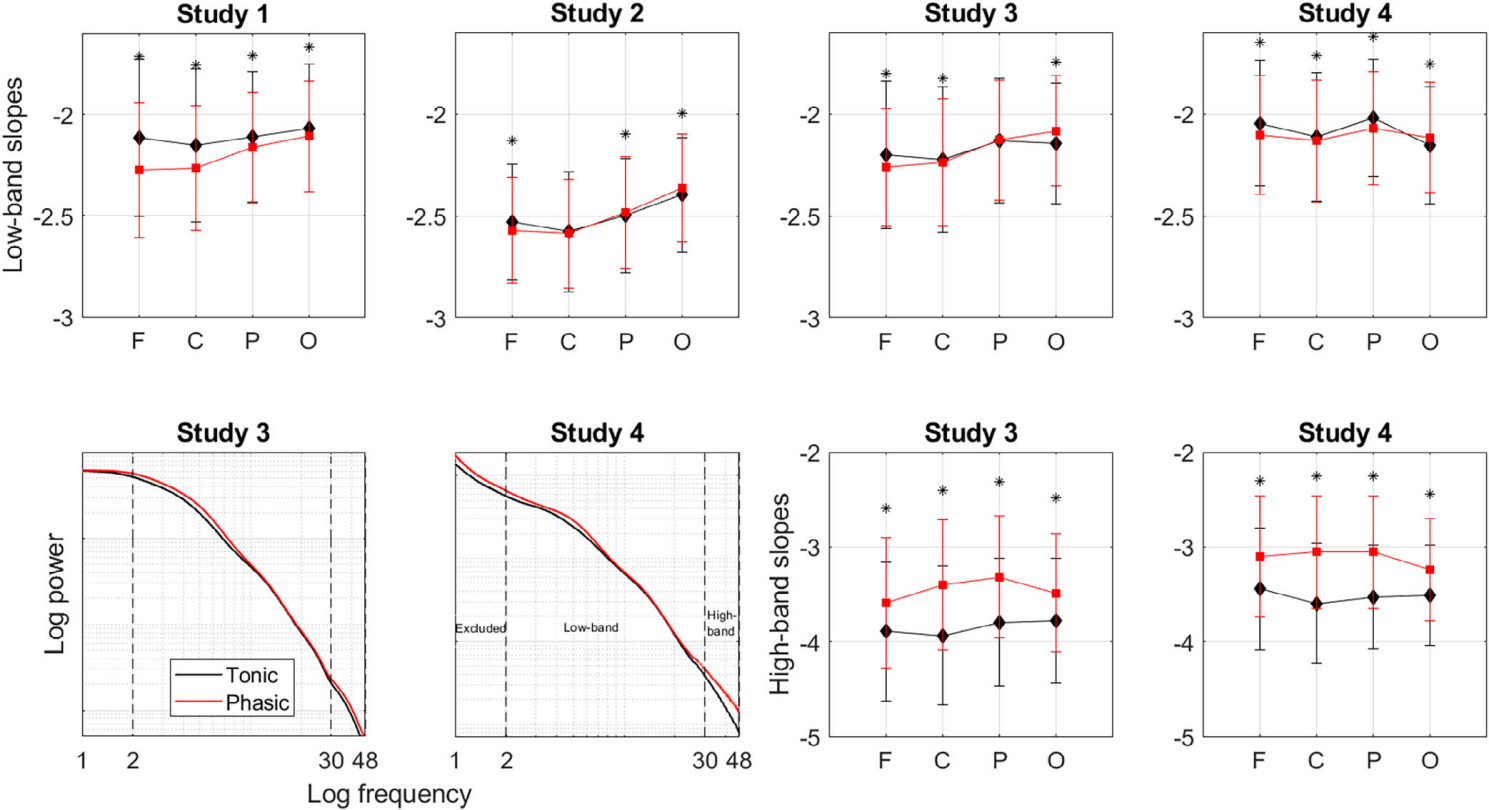
Epoched data. Aperiodic activity during tonic and phasic states. First row: slopes of the aperiodic power component in the low‐band averaged over the phasic versus tonic epochs over each topographical area separately for Datasets 1–4 show some topographical variation in the strength and direction of the state differences across the datasets. Second row, right: slopes in the high‐band for Datasets 3–4. The tonic state (black diamonds) shows steeper high‐band aperiodic slopes compared with the phasic state (red squares) over all topographical areas in both datasets. *Statistically significant difference between tonic and phasic states (*p* < 0.05); F, frontal; C, central; P, parietal; O, occipital. Second row, left: aperiodic component of spectral power averaged over frontal electrodes plotted as a function of frequency for tonic (black line) versus phasic (red line) states of REM sleep in Datasets 3–4. Shading indicates standard errors of the mean. The phasic state shows steeper decay of the aperiodic component in the low‐frequency band (2–30 Hz) and flatter decay in the high‐frequency band (30–48 Hz) compared with the tonic state. Activity < 2 Hz was not analysed. REM, rapid eye movement.

We analysed the data merged from four datasets into one large dataset as well as each dataset individually. For the former analysis, we transformed the aperiodic slopes into *z*‐scores as when we pooled together the data acquired by different devices, we detected significant differences between Dataset 2 and the rest of the datasets (Figure 1). Specifically, for each participant, we merged the values calculated for the four topographical areas of the phasic and tonic states into one vector and then performed *z*‐transformation. After the *z*‐transform, we averaged a given variable over each area over each REM state separately. Nevertheless, given that the raw values of spectral exponents/slopes have their own functional significance (as mentioned in more detail in Discussion), we also analysed each dataset individually while using raw values of aperiodic slopes. Raw slope values are reported in the Excel File on https://osf.io/zhyf7.

### 2.4 Statistical analysis of the epoched data

To compare aperiodic slopes during phasic versus tonic states, we used the ANOVA with the four‐level “brain area” and two‐level “REM sleep state” as within‐subject factors. We performed ANOVAs for high‐ and low‐frequency bands separately with Benjamini–Hochberg's adjustment to control for multiple (two) comparisons with a false discovery rate set at 0.05 and the *α*‐level set in the 0.025–0.050 range. We applied Greenhouse–Geisser's correction as Mauchly's test revealed that the sphericity assumption was violated (*ε* < 0.75, *p* < 0.05). The assumptions of normality and homogeneity of variance were tested using the Q–Q plot and Levene's homogeneity test, respectively. Then, we performed post hoc analyses to compare binary phasic and tonic states for each topographical area separately using two‐tailed Student's paired *t*‐tests. Effect sizes were calculated with Cohen's *d*.

To assess the ability of the aperiodic slopes to discriminate between phasic and tonic states of REM sleep at an individual level, we calculated the area under the receiver operating characteristic (ROC) curve (AUC) for each topographical area. Benjamini–Hochberg's adjustment was applied to control for multiple comparisons (eight tests corresponding to four areas for low‐ and high‐bands separately) with a false discovery rate set at 0.05 and the *α*‐level set in the 0.0063 (i.e. 0.05/8)–0.0500 range. The sensitivity and specificity of the tests were evaluated using the median of all values (both phasic and tonic) of a given area as a cutoff. For the ROC and ANOVA, we used SPSS software (version 25; SPSS).

### 2.5 EM quantification

To control for a possible bias of binary categorization of REM sleep into phasic and tonic states, we also used a continuous approach to define phasic oculomotor activity across the duration of REM sleep episodes. For this exploratory analysis, we used EOG data from Dataset 3 (*n* = 20) and Dataset 4 (*n* = 40) filtered in the 0.3–10‐Hz range. We applied to it the *rem_detect* function of the YASA package for Python (Vallat & Walker, 2021) using its defaults (minimum amplitude: 50 μV; duration: 0.3–1.2 s; rapid EMs frequency: 0.5–5 Hz). As an outcome measure, we used the absolute peak amplitude of rapid EMs. This variable focuses on the EM morphology in relation to surrounding signal activity, ensuring that the identified peak is representative of an EM event rather than an isolated amplitude spike (Vallat & Walker, 2021).

We checked the EM detection by YASA via a visual inspection of the continuous EOG data. Likewise, we manually calculated the peak value of rapid EMs by identifying the maximum amplitude within a predefined 4‐s window based on raw amplitude measurements. The coefficients of correlation between the amplitude calculated by YASA and that calculated manually was 0.87 for Dataset 3 and 0.90 for Dataset 4. An in‐depth analysis of one participant further showed that the percentage of agreement between the YASA and visual inspection (calculated as true positives + true negatives) equalled 91%. The percentage of rapid EMs missed by the YASA (false negatives) equalled 0.25%. The percentage of rapid EMs missed by manual coding (false positives) equalled 8.69%. Then, we correlated EM amplitudes with aperiodic slopes to obtain Spearman correlation coefficients.

### 2.6 Temporal relationships between EMs and aperiodic slopes

To assess temporal relationships between oculomotor and aperiodic activities across the duration of a single REM sleep episode, we cross‐correlated between the time series of EM amplitudes defined with continuous EOG (as described in the previous section) and the time series of aperiodic slopes calculated using continuous preprocessed EEG. Namely, preprocessing included filtering and the removal from the EEG signal independent component(s) related to EMs as described in Simor et al. (2019). Then, we calculated aperiodic slopes as described above. To approximate the distribution of the measured values to the normal distribution, both time series were ranked using the *tiedrank* function and correlation coefficients were transformed to Fisher *z*‐scores. Confidence intervals of 95% were calculated to infer statistical significance. Cross‐correlations were performed for the lags (i.e. temporal intervals between the two time series) lying between −5 and 5 min with a step of 4 s for low‐ and high‐frequency bands for each topographical area and for each REM sleep episode separately.

A previous EOG study noted that EM density fluctuates at about 2‐min intervals (Ktonas et al., 2003). Therefore, we included only episodes longer than 10 min to obtain the time series of sufficient length that, theoretically, would contain several periods of phasic bursts, and thus sufficient statistical power. Likewise, this selection increased the homogeneity of the analysed episodes as early‐night REM sleep episodes (which have been mainly excluded after this selection) are usually both considerably shorter and qualitatively different from the late‐night ones because of circadian and homeostatic effects (Simor et al., 2023). This procedure resulted in 40 REM sleep episodes from 19 participants (in one participant, all REM sleep episodes were shorter than 10 min) that on average lasted for 17.7 ± 9.4 min from Dataset 3, and 83 episodes from 36/40 participants (in four participants, all REM sleep episodes were < 10 min) that lasted for 16.7 ± 7.2 min from Dataset 4.

Having performed cross‐correlation at the individual episode level, next we evaluated its episode‐centred effect sizes to better understand the dynamics across all REM sleep episodes. For this, we counted the number of significant correlations between the two time series and divided it by the total number of all (significant and non‐significant) performed correlations. Finally, we assessed the population prevalence of the person‐centred effect sizes with the Bayesian prevalence. This method estimates the proportion of the population that would show the effect if they were tested in this experiment (Ince et al., 2022). As an output, it provides the maximum *a posterior* estimate – the most likely value of the population parameter and the highest posterior density intervals – the range within which the true population value lies with the specified probability level of 96%. To perform this analysis, we used an online web application available at https://estimate.prevalence.online.

## 3 RESULTS

### 3.1 Epoched data

The phasic state of REM sleep showed steeper (i.e. more negative) slopes of the aperiodic power component in the low (2–30 Hz) band compared with the tonic state with some topographical variation across the datasets. Repeated‐measures ANOVA performed for the pooled dataset (*n* = 95) revealed a significant effect of the area (*F*_92,3_ = 22), state (*F*_94,1_ = 23) as well as the state–area interaction (*F*_92,3_ = 72; all *p* < 0.001). The ROC analysis revealed that the low‐band slopes measured over the frontal (AUC = 0.77 ± 0.03, *p* < 0.001), central (AUC = 0.64 ± 0.04, *p* = 0.001) and parietal (AUC = 0.71 ± 0.04, *p* < 0.001) areas can discriminate between the two states.

Post hoc analysis performed for each dataset separately further showed that for Dataset 1, steeper phasic slopes could be observed over all areas with an anterior‐to‐posterior gradient of the strength of effect sizes with the largest difference measured over the frontal area. For Dataset 4, the effect could be observed over the frontal, central and parietal areas. For Dataset 3, the effect could be observed over the frontal and central areas. For Dataset 2, the effect could be observed over the frontal area only. Interestingly, in Datasets 2–4, aperiodic slopes over the occipital area were steeper during tonic versus phasic states, while in Dataset 1 we observed the opposite effect (Figure 1; Table 2). The effect sizes were small.

**TABLE 2:** Aperiodic slopes.

Frequency	Dataset	Area	Frontal	Central	Parietal	Occipital
Low‐band (2–30 Hz)	Dataset 1 (2040 versus 2039 epochs, 20 participants)	Tonic	−2.12 ± 0.39	−2.15 ± 0.38	−2.11 ± 0.32	−2.07 ± 0.32
		Phasic	−2.28 ± 0.33	−2.27 ± 0.31	−2.16 ± 0.27	−2.11 ± 0.27
		Effect size, *d*	0.446	0.322	0.177	0.135
		*t*‐test, *p* <	0.001	0.001	0.001	0.001
	Dataset 2 (4994 versus 5934 epochs, 17 participants)	Tonic	−2.53 ± 0.28	−2.58 ± 0.30	−2.50 ± 0.28	−2.40 ± 0.28
		Phasic	−2.57 ± 0.26	−2.59 ± 0.27	−2.49 ± 0.28	−2.36 ± 0.26
		Effect size, *d*	0.114	0.013	−0.050	−0.119
		*t*‐test, *p*	< 0.001	0.494	0.010	< 0.001
	Dataset 3 (6538 versus 6168 epochs, 18 participants)	Tonic	−2.20 ± 0.36	−2.22 ± 0.36	−2.13 ± 0.31	−2.14 ± 0.30
		Phasic	−2.26 ± 0.29	−2.24 ± 0.31	−2.13 ± 0.30	−2.08 ± 0.27
		Effect size, *d*	0.188	0.040	−0.009	−0.214
		*t*‐test, *p*	< 0.001	0.025	0.615	< 0.001
	Dataset 4 (6653 versus 8149 epochs, 40 participants)	Tonic	−2.04 ± 0.31	−2.11 ± 0.32	−2.02 ± 0.29	−2.15 ± 0.29
		Phasic	−2.10 ± 0.29	−2.13 ± 0.30	−2.07 ± 0.28	−2.11 ± 0.27
		Effect size, *d*	0.193	0.066	0.181	−0.140
		*t*‐test, *p* <	0.001	0.001	0.001	0.001
High‐band (30–48 Hz)	Dataset 3	Tonic	−3.89 ± 0.73	−3.94 ± 0.73	−3.79 ± 0.68	−3.78 ± 0.66
		Phasic	−3.59 ± 0.69	−3.40 ± 0.69	−3.32 ± 0.64	−3.49 ± 0.62
		Effect size, *d*	−0.417	−0.750	−0.723	−0.456
		*t*‐test, *p* <	0.001	0.001	0.001	0.001
	Dataset 4	Tonic	−3.44 ± 0.65	−3.60 ± 0.63	−3.53 ± 0.55	−3.51 ± 0.53
		Phasic	−3.10 ± 0.64	−3.06 ± 0.60	−3.06 ± 0.60	−3.24 ± 0.54
		Effect size, *d*	−0.532	−0.882	−0.817	−0.506
		*t*‐test, *p* <	0.001	0.001	0.001	0.001

In addition, in Dataset 3 (*n* = 18) and Dataset 4 (*n* = 40) we could perform the analysis in the high‐band (30–48 Hz). We found that the phasic state showed flatter (more positive) high‐band slopes compared with the tonic state over all areas with moderate‐to‐large effect sizes in both datasets. The repeated‐measures ANOVA in pooled Datasets 3 and 4 (*n* = 58) revealed a significant effect of the area (*F*_55,3_ = 4, *p* = 0.013), state (*F*_57,1_ = 557, *p* < 0.001) as well as the state–area interaction (*F*_55,3_ = 56, *p* < 0.001). The ROC analysis revealed that the high‐band slopes measured showed very high sensitivity and specificity in discriminating between REM states, especially over the central (AUC = 0.982 ± 0.014, *p* < 0.001) and parietal (AUC = 0.996 ± 0.003, *p* < 0.001) areas.

### 3.2 Continuous data: between‐episode analysis

**FIGURE 2:**
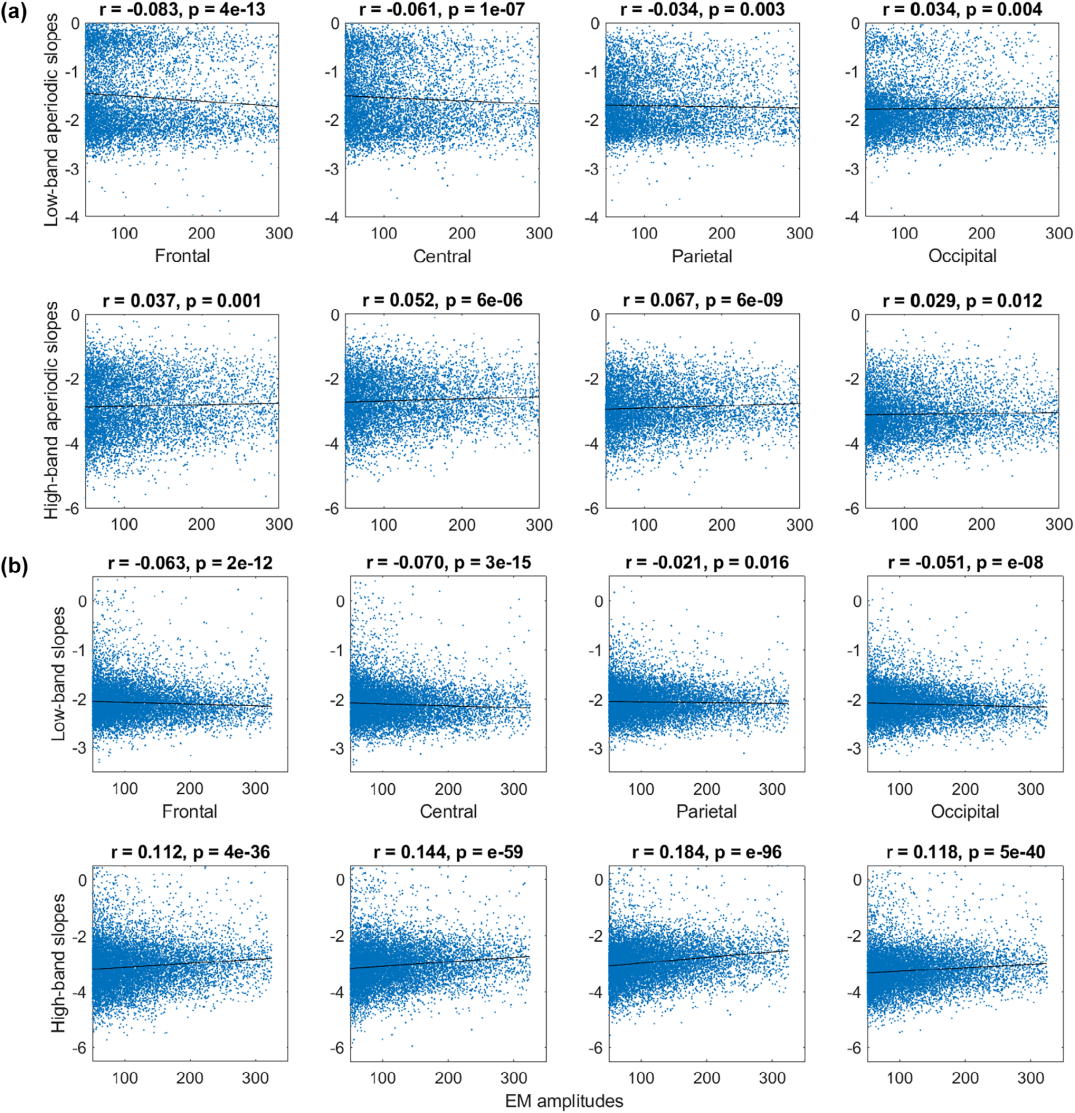
Continuous data: between‐episode analysis. EM amplitudes versus aperiodic slopes in the low (2–30 Hz, upper rows) and high (30–48 Hz, lower rows) bands over frontal, central, parietal and occipital electrodes from the continuous data from Dataset 3 (b, 20 participants, 9267 epochs) and Dataset 4 (c, 40 participants, 12,588 epochs). Each dot represents a 4‐s epoch, the data of all participants is pooled. In both datasets, EM amplitudes correlated negatively with low‐band slopes over frontal, central and parietal areas (first rows in a and b), and positively with the high‐band aperiodic slopes over all areas (second rows in a and b). EM, eye movements; *r*, Spearman's correlation coefficient.

**FIGURE 3:**
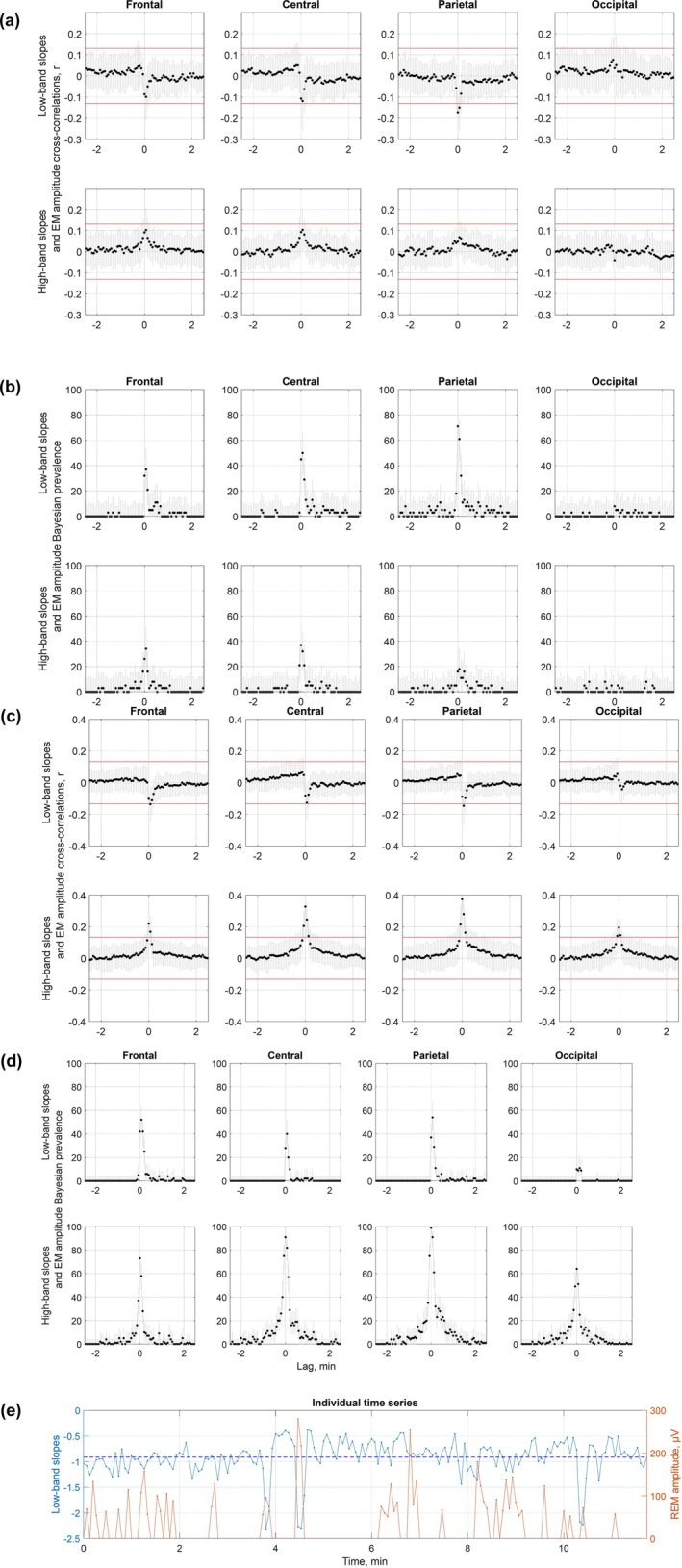
Continuous data: within‐episode analysis. (a, c) Average coefficients of cross‐correlations with standard deviations. Cross‐correlations were calculated between the ranked time series of EM amplitudes and low‐band (top) or high‐band (bottom) aperiodic slopes averaged over frontal, central, parietal and occipital electrodes for each REM sleep episode individually in Dataset 3 (a) and Dataset 4 (c). Correlation coefficients were Fisher *z*‐transformed and averaged over all episodes. Negative and positive lags mean that aperiodic slope time series are leading and lagging, respectively. The horizontal red lines mark the confidence interval of 95%, and the absolute values above these lines indicate statistical significance (|*r*| > 0.1, *p* < 0.05). In Dataset 3 (a), EM time series correlated negatively with low‐band aperiodic slopes over the parietal area at 0 and 4‐s lags only. In Dataset 4 (c), EM time series correlated negatively with low‐band slopes over the frontal and parietal areas at 4‐s lags (first row) and positively with high‐band slopes at ~ −4‐s to 4‐s lags over all areas (second row). (b, d) Bayesian population prevalence of effect sizes. The Bayesian population prevalence (in %) of the episode‐centred effect sizes (black dots), i.e. the most likely value of the population parameter, and the highest posterior density intervals (grey lines), the range within which the true population value lies with the probability level of 96%. Episode‐centred effect sizes were obtained by counting the number of significantly negative (for low‐band, top) or positive (for high‐band, bottom) correlations between time series of aperiodic slopes and EM amplitudes and dividing it by the total number of all correlations (both significant and non‐significant) for Dataset 3 (b, *n* = 40) and Dataset 4 (d, *n* = 83). (e) Representative time series. The time series of EM amplitudes and low‐band parietal aperiodic slopes calculated for each 4‐s epoch during a 15.4‐min REM sleep episode. The horizontal dashed blue line shows the slope mean. Higher EM amplitudes are associated with steeper slopes at the time of the event (*r* = −0.31, *p* < 0.001). EM, eye movement; REM, rapid eye movement.

Besides the binary REM sleep categorization described above, we also used the continuous approach to assess REM sleep heterogeneity. Namely, we quantified EM amplitudes and correlated them with aperiodic slopes at the between‐episode (this section; Figure 2) and within‐episode (next section; Figure 3) levels.

For between‐episode analysis, we pooled together the epochs with EM events (defined as EOG amplitude > 50 μV) – this resulted in 9,267 4‐s epochs from 20 participants in Dataset 3, and 12,588 4‐s epochs from 40 participants in Dataset 4. We found that EM amplitudes correlated negatively with the low‐band aperiodic slopes (calculated from the EEG of the corresponding epochs) over the frontal, central and parietal areas in both datasets. In addition, EM amplitudes and low‐band occipital aperiodic slopes showed a positive correlation in Dataset 3 and a negative correlation in Dataset 4. EM amplitudes correlated positively with the high‐band aperiodic slopes over all areas in both datasets (Figure 2).

Interestingly, we observed that aperiodic slopes derived from the continuous data distributed bimodally in Dataset 3 but not in Dataset 4. Slopes derived from the epoched data showed unimodal distribution in all four datasets (shown and discussed in Figure S2).

### 3.3 Continuous data: Within‐episode analysis

Next, we assessed temporal relationships between aperiodic activity and EMs within each REM sleep episode using cross‐correlations. An example time series of EM amplitudes and aperiodic slopes for a representative REM sleep episode is shown in Figure 3. All REM episodes are presented in Supporting Information pdf file on https://osf.io/zhyf7.

We found that time series of EM amplitudes and low‐band aperiodic slopes correlated negatively for 0 and 4‐s lags over the parietal area in Dataset 3 (Figure 3a, first row), and for 4‐s lags over the frontal and parietal areas in Dataset 4 (Figure 3c, first row). At the individual episode level, these correlations were observed in 60%–72.5% of the analysed episodes in Dataset 3 (Figure 3b), and in 52%–54% of the episodes in Dataset 4 (Figure 3d).

Interestingly, time series of EM amplitudes and high‐band aperiodic slopes over all areas correlated positively for 0–4‐s lags over frontal areas, for −4 to 8‐s lags over central and parietal areas, and for −4 to 4‐s lags over occipital areas in Dataset 4 (Figure 3c, second row) but not in Dataset 3. For zero‐lags, high‐band aperiodic slopes correlated with EM amplitudes in 64%–100% of the episodes in Dataset 4 (Figure 3d, second row), and in 10%–42.5% of the episodes only in Dataset 3 depending on the topographical area (Figure 3b, second row). For the rest of the time lags, aperiodic activity preceding or following EM events did not cross‐correlate with EM amplitudes in both datasets. Specifically, less than 15% of all REM sleep episodes showed significant correlations for these time lags, indicating that rapid Ems could not be predicted by the magnitude of aperiodic slopes and vice versa.

## 4 DISCUSSION

This study explored how REM sleep states differ with respect to aperiodic neural activity using either binary phasic‐tonic or continuous (EM quantification) approach to assess REM sleep heterogeneity. Both approaches showed similar results for instantaneous measurements at the time at or close to an EM event. Namely, EM amplitudes/phasic states correlated negatively with low‐band aperiodic slopes and positively with high‐band aperiodic slopes with some topographical variation across datasets. The temporal analysis within individual REM sleep episodes revealed that Ems could not be predicted by preceding aperiodic activity or vice versa.

### 4.1 Slope value meaning

The REM sleep is rather heterogeneous, with its tonic constituents being depicted as more wake‐like or intermediate states between wakefulness and phasic REM sleep concerning several characteristics, including environmental alertness and external information processing (Simor et al., 2020). The current study supports this point of view from a new angle, that of aperiodic neural activity.

Aperiodic activity is described by the spectral exponent *α* that reflects the steepness of the decay of the power‐law function 1/f^α^ (He, 2014). In the log–log space, exponents are equivalent to slopes, the variable reported in this study. Crucially, specific ranges of spectral exponents define specific features of the underlying time series (Bódizs et al., 2024) as follows.

Exponents recorded during wake tend to be closer to fractional values around −1 (f^−1^ or, equivalently, 1/f) or slightly above as derived from the broadband fitting. An exponent of −1 is a sign of anti‐persistent dynamics characterized by substantial differences between time scales and negative correlations between successive increments of time series (e.g. amplitude decreases are followed by their increases and vice versa). Nevertheless, the nominal exponent values during wake vary substantially and could be as low as −2, especially for wake after sleep onset (Schneider et al., 2022).

The power‐law function depicting NREM sleep exhibits fractional exponents between −3 and −2.5 (f^−3^–f^−2.5^; see table 3 in Bódizs et al., 2024 for benchmarks). These exponents are the sign of persistent dynamics, where serial increments correlate positively (i.e. increases within the time series are followed by increases and vice versa) and overall neural dynamics differ less when observed across different time scales.

The REM sleep signals show fractional exponents of about −2 (f^−2^), which is characteristic of the time series with rather independent successive increments. Importantly, *α* = −2 is seen as a critical value delimiting evident wakefulness and sleep or sleepiness (Bódizs et al., 2024).

In four different datasets of the current study, average exponent/slope values recorded during REM sleep ranged from −2.0 to −2.6 within the fitting band of 2–30 Hz. This is in line with Schneider et al. (2022) who reported REM sleep exponent values of −2.4 for the fitting band of 2–48 Hz in young adults. The observed numbers reveal some overlap with fig. 4 in Miskovic et al. (2019) who show that the slope values measured during REM sleep are distributed from −2 to −1 for the 0.5–35‐Hz fitting band.

Our study further specified that tonic states showed average slopes ranging from −2.1 to −2.5, while phasic slopes presented with values from −2.3 to −2.6 (Table 2; see also Figure S2 showing histograms). Thus, concerning the spectral exponent range, tonic states are intermediate between phasic REM sleep and wakefulness. This is in coherence with a reported wake‐like nature of the tonic state regarding frequency‐specific cortical synchronization (Simor et al., 2018) or auditory stimulation effects as quantified by event‐related potentials (Takahara et al., 2006).

### 4.2 Topography

It has been suggested that time series showing higher anti‐persistency are associated with a more disorganized/randomized network that favours environmental responsiveness during wakefulness, and possibly reflect high turbulence/fluctuations in neuronal activity (Lina et al., 2019). Interestingly, aperiodic dynamics showing more anti‐persistency (flatter aperiodic slopes) are often described as more complex. A recent study showed that tonic states present with higher signal complexity compared with phasic states (Lu et al., 2024). This is in coherence with previous reports on correlations between aperiodic slopes and Lempel–Ziv complexity during NREM, REM sleep and rest (Höhn et al., 2024; Medel et al., 2023; Rosenblum, Shiner, et al., 2023).

Seen in the context of REM sleep states, higher anti‐persistency/complexity of the low‐band signal recorded during tonic states over frontal areas seen across all datasets could be attributed to higher levels of conscious content that require more complex brain activity. This is in line with the more wake‐like and “online” mode of the tonic state (Simor et al., 2020) as well as with the nature of dream reports obtained after tonic periods. Specifically, “tonic” dreams have been characterized by the presence of thinking, recognizing, interpreting, being aware, comparing and/or explaining, overall, being more thought‐like than the visual “phasic” dreams (Molinari et al., 1969).

Interestingly, here, in 3/4 datasets, low‐band occipital aperiodic slopes were flatter in phasic compared with tonic states, opposite to that measured over more anterior areas. This resembles the established link between the posterior cortical area‐specific decrease in slow‐wave activity and the presence of conscious experience in the form of dreams (Siclari et al., 2017). Intriguingly, in mice, neural activity in the occipital cortical regions (including visual areas) gradually increases during NREM–REM sleep transitions and stays high throughout the entire REM episode (Wang et al., 2022).

### 4.3 Continuous heterogeneity of REM sleep

Though binary REM sleep categorization is broadly used in literature, one should keep in mind that phasic and tonic states might, alternatively, represent “ends of a continuum” (Bueno‐Junior et al., 2023). Another view is that REM sleep is “the sequential occurrence of different activity patterns during prolonged tonic activations punctuated by phasic bursts” (Tononi et al., 2024). An earlier view states that REM sleep is unified in its tonic background characteristics (such as low‐voltage mixed frequency EEG and EMG suppression) with superimposed phasic events occurring heterogeneously (Molinari et al., 1969). Bueno‐Junior et al. (2023) understand REM sleep as a heterogeneous structure where some signals show binary states (e.g. facial and oculomotor activity in both mice and humans), whereas others show continuous pattern (e.g. theta frequency in mice and respiration rate in humans). Intriguingly, the authors further suggest that binary and continuous features co‐occur on an infra‐slow timescale (< 0.1 Hz; Bueno‐Junior et al., 2023). Following this, here, besides the tonic‐phasic dichotomy, we also assessed REM sleep heterogeneity using the continuous approach that quantifies EM amplitudes.

We found that steeper low‐band parietal aperiodic slopes at or close to the time of an EM event were associated with higher EM amplitudes in 52%–70% of the analysed REM sleep episodes in both tested datasets. Flatter high‐band aperiodic slopes over all areas at or close to the time of an EM event were associated with higher EM amplitudes in 64%–100% of the episodes in Dataset 4, yet only in 10%–42.5% of the episodes in Dataset 3, meaning that further replication is needed.

The analysis of temporal, intra‐REM sleep episode relationships between aperiodic and oculomotor activities revealed that EMs could not be predicted by preceding (> 4 s) aperiodic activity or vice versa. A possible interpretation of this finding is that aperiodic and oculomotor activities are regulated by a common (e.g. brainstem) pacemaker. Notably, rapid EMs coincide with the ponto‐geniculo‐occipital circuitry, which could not be measured in humans with non‐invasive techniques (Simor et al., 2020; however, see Wehrle et al., 2005).

The temporal aspects of the phasic activity of REM sleep, its neural correlates and functional significance are far from being established. Among others, it has been suggested that EMs represent time points at which neural activity and associated visual‐like processing is updated, possibly reflecting a change of the visual imagery in dreams (Andrillon et al., 2015). Likewise, EMs have been linked to consolidation of procedural memory involving novel cognitive strategies and problem‐solving skills, where EMs correlate with theta and sensorimotor rhythms (van den Berg et al., 2023). Another view is that EM density reflects neural excitation as discussed in more detail in the next section.

### 4.4 Excitation‐to‐inhibition balance and aperiodic activity

Besides the low‐band (2–30 Hz) aperiodic activity, in 2/4 datasets, we also measured high‐band (30–48 Hz) activity as the latter is considered to reflect the balance between excitatory and inhibitory neural currents (Gao et al., 2017). This, in turn, defines a specific arousal state and a conscious experience of an organism. We observed flatter high‐band aperiodic slopes during phasic versus tonic states over all areas in both tested datasets (Figure 1) with very high sensitivity and specificity. This finding was confirmed by the continuous approach in one out of two tested datasets. Seen in the light of Gao's theory, our findings suggest that the phasic state could be associated with a relative shift towards neural excitation. This is also in line with the known increase in gamma‐band power during the phasic state (Simor et al., 2019). Increases in both high‐band aperiodic activity and gamma power seen during phasic states probably have the same neural origin, reflecting increases in cortical excitability. This may produce short bursts of consciousness, resulting in dreams with vivid visual imagery and high emotional and perceptual content (Simor et al., 2020; Usami et al., 2017). Our findings showing that higher EM amplitudes coincide with flatter high‐band aperiodic slopes (that reflect a shift towards neural excitation) are also in line with the view on EM density as a reflection of the build‐up of a pressure to awaken across a night. Indeed, it has been shown that EM density increases across sleep cycles (while sleep pressure, need and depth decrease) and is higher at the circadian time of higher arousal (van den Berg et al., 2023).

### 4.5 Limitations, strengths and conclusions

The major limitation of this study is its cross‐sectional observational design and correlational approach, and thus an inability to shed light on the mechanism underlying the observed effects. The strengths of this study include its sample size, scripts and data sharing and self‐replication in four independent datasets for the epoched low‐band data and two datasets for the epoched high‐band and continuous data. While the findings on low‐band aperiodic slopes were robust and replicable over all datasets for the frontal areas, the rest of the areas showed some heterogeneity across datasets (Figure 1). Likewise, the findings of the continuous within‐episode high‐band slope analysis were observed in one out of two datasets (Figure 3). This might stem from the fact that all datasets have idiosyncrasies due to differences in experimental settings, design and context, data acquisition and participant inclusion criteria, as well as subject sampling error, non‐representative subjects, too high variability between them, undetected artefacts, arousals, statistical false alarms or other limitations (Cohen, 2014). Likewise, topographical differences might result from a difference in reference used (Table 1) as different reference electrodes may highlight some (and not other) effects in different magnitudes. Even if all datasets used the same reference, minor differences in electrode positions and noise in the reference electrodes could cause subtle differences. Finally, the EEG spatial resolution is not precise due to volume conduction (Cohen, 2014).

In conclusion, our study demonstrates that aperiodic neural activity can serve as a neural correlate and a reliable marker able to differentiate between phasic and tonic constituents of REM sleep, and reflects the intensity of phasic oculomotor events, providing further evidence on the heterogeneity of REM sleep.

## AUTHOR CONTRIBUTIONS

**Yevgenia Rosenblum:** Conceptualization; writing – original draft; methodology; visualization; writing – review and editing; formal analysis; supervision; investigation; validation; project administration; data curation. **Tamás Bogdány:** Data curation; validation; investigation. **Lili Benedikta Nádasy:** Investigation; data curation. **Xinyuan Chen:** Investigation; data curation. **Ilona Kovács:** Writing – review and editing; investigation; data curation. **Ferenc Gombos:** Writing – review and editing; investigation; data curation. **Péter Ujma:** Writing – review and editing; investigation; data curation. **Róbert Bódizs:** Writing – review and editing; data curation; investigation. **Nico Adelhöfer:** Writing – review and editing; supervision. **Péter Simor:** Writing – review and editing; conceptualization; investigation; funding acquisition; data curation; supervision; resources; project administration. **Martin Dresler:** Writing – review and editing; supervision; funding acquisition; resources; investigation.

## CONFLICT OF INTEREST STATEMENT

The authors declare no competing interests.

## Supporting information


**DATA S1.** Supporting Information.

## Data Availability

The data that support the findings of this study are available in osf at https://osf.io. These data were derived from the following resources available in the public domain: ‐ Interoception in REM microstates, https://osf.io/2vptx ‐ REM phasic vs tonic, https://osf.io/9k5hb.
