# Supplementary material for: Aperiodic neural activity distinguishes between phasic and tonic REM sleep
Source: J Sleep Res. 2024 Dec 26;34(4):e14439. doi: 10.1111/jsr.14439 (PMC12215217; doi:10.1111/jsr.14439)
Supplement: Supplementary file 1 — DATA S1. Supporting Information. [file JSR-34-e14439-s001.docx]

**Aperiodic neural activity distinguishes between phasic and tonic REM sleep**

Yevgenia Rosenblum ^1^, Tamás Bogdány ^2, 3^, Lili Benedikta Nádasy ^2^, Xinyuan Chen ^1^, Ilona Kovács ^4^, Ferenc Gombos ^4, 5^, Péter Ujma ^6^, Róbert Bódizs ^6^, Nico Adelhöfer ^1 *^, Péter Simor ^2, 6 *^, Martin Dresler ^1 *^

^1^ Radboud University Medical Centre, Donders Institute for Brain, Cognition and Behavior, Nijmegen, Netherlands,

^2^ Institute of Psychology, ELTE Eötvös Loránd University, Budapest, Hungary,

^3^ Doctoral School of Psychology, ELTE, Eötvös Loránd University, Budapest, Hungary

^4^ HUN-REN-ELTE-PPKE Adolescent Development Research Group, Faculty of Education and Psychology, Eötvös Loránd University, Budapest, Hungary,

^5^ Pázmány Péter Catholic University, Department of General Psychology, Budapest, Hungary,

^6^ Semmelweis University, Institute of Behavioural Sciences, Budapest, Hungary.

* - equal contribution.

Corresponding author: Yevgenia Rosenblum, e-mail: yevgenia.rozenblum@donders.ru.nl

#

# **Supplementary Material**

Contents

[**Supplementary Material** 1](#_Toc182348036)

[**Supplementary Figure 1. Epoched data. Aperiodic activity during tonic and phasic states in the 5 – 30Hz band.** 2](#_Toc182348037)

[**Frequency distribution of aperiodic slopes** 3](#_Toc182348038)

[**Supplementary Figure 2. Epoched and continuous data. Slope frequency distribution.** 6](#_Toc182348039)

[**Supplementary Figure 3. Continuous data of Dataset 3: between-episode analysis.** 8](#_Toc182348040)

[**Electrocardiography (ECG)** 8](#_Toc182348041)

## **Supplementary Figure 1. Epoched data. Aperiodic activity during tonic and phasic states in the 5 – 30Hz band.**

Slopes of the aperiodic power component in the 5 – 30 Hz for Datasets 1 – 4 averaged over the phasic vs tonic epochs over each topographical area separately. The analysis in this band was performed to control for possible residual contamination of the EEG signal by eye movement events. Steeper phasic (red squares) vs tonic (black diamonds) slopes could be observed over frontal, central and parietal areas in Datasets 1 and 4, over frontal and central areas in Dataset 2 and over the frontal area only in Dataset 3. Flatter phasic vs tonic slopes could be observed over parietal and occipital areas in Datasets 2 and 3 and over occipital area in Dataset 4. * – statistically significant difference between tonic and phasic states (p < 0.05), F – frontal, C – central, P – parietal, O – occipital.

## **Frequency distribution of aperiodic slopes**

We observed that aperiodic slopes derived from the continuous data of Dataset 3 distributed bimodally (Fig.S2 E), e.g., the frontal low-band slope distribution had a major mode (i.e., the larger local peak) centered at -2.1 (the slope value typical for REM sleep) and the minor mode centered at -0.4 (a value more typical for the wake, see also Discussion, section *Spectral exponent range interpretation*). Slope distribution for each participant is presented in Fig.S2 G, showing that 5 out of 20 participants show bimodal distribution.

At the same time, slope distribution of the continuous data from Dataset 4 (Fig.S2 F) as well as that of the epoched data from all datasets (Fig.S2 A – D) was unimodal. Given that the bimodal distribution was observed in only one dataset, we hypothesize that it probably reflects idiosyncrasies of that dataset that might stem from subject sampling error, non-representative subjects, too high variability, undetected artifacts, arousals as authentic elements of undisturbed sleep microstructure, statistical false alarms or other limitations (Cohen, 2014).

The finding on the bimodal distribution of the low-band aperiodic slopes raised the question of whether the correlations between aperiodic slopes and EM amplitudes observed in Dataset 3 are rooted in this bimodality. To control for the heterogeneity of the continuous data of Dataset 3, we further stratified all slopes into two groups that would be distributed unimodally. The cutting points were chosen both theoretically and empirically. The first cutting point was equal to -1, where slopes < -1 were considered typical for sleep and sleepiness (including wake after sleep onset) and slopes > -1 were considered typical for evident wakefulness (Bódizs et al., 2024). The second cutting points were data-driven, i.e., we cut the data at the antimodes -1.3 and -1.5.

We found that the direction, topography and significance of the correlations between EM amplitudes and slopes < -1 remained the same as for all slopes (i.e., before the stratification). Namely, EM amplitudes correlated negatively with the low-band aperiodic slopes over the frontal, central and parietal areas and positively with the low-band aperiodic slopes over the occipital area (Fig.S3 A). Similar pattern was observed when we calculated the slopes in the 5 – 30 Hz band to control for possible residual contamination of the EEG signal by EMs (Fig.S3 B).

.

**B: D2, epoched**

**A: D1, epoched**

**C: D3, epoched**

**E: D3, continuous**

**D: D4, epoched**

`


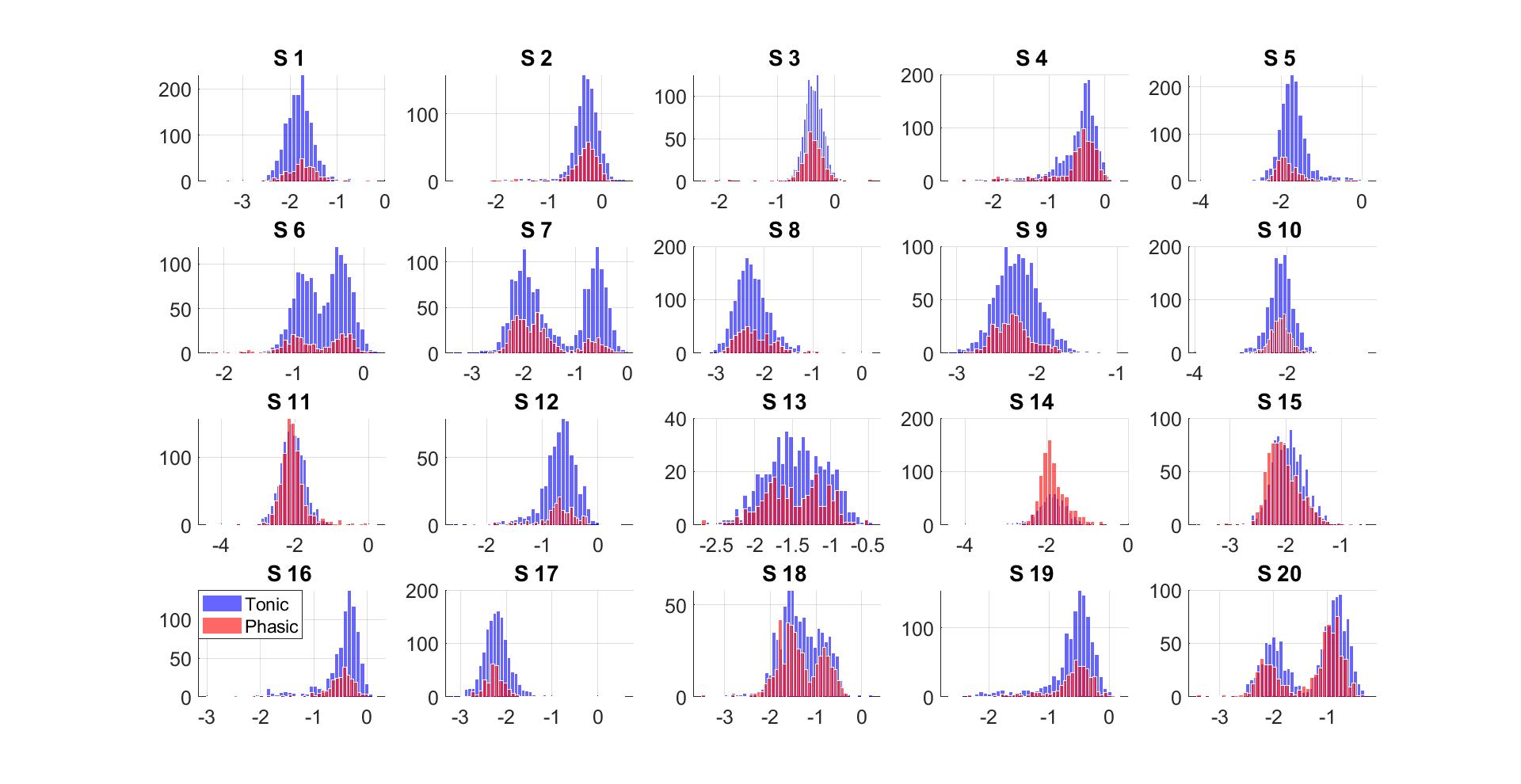


**G: D3, continuous**

**F: D4, continuous**

## **Supplementary Figure 2. Epoched and continuous data. Slope frequency distribution.**

Distribution of aperiodic slopes in the low (2 – 30 Hz, all datasets) and high (30 – 48Hz, Dataset 3 – 4 only) bands over frontal, central, parietal and occipital electrodes of the epoched data from Datasets 1 – 4 (A – D) and from the continuous data from Datasets 3 – 4 (E – F). Only the continuous data from Dataset 3 shows bimodal distribution (E), e.g., frontal low-band slope distribution has a major mode centered at -2.1 (the slope values typical for REM sleep), and a minor mode centered at -0.4 (the slope values typical for wakefulness). The rest of the datasets (A – D, F) shows unimodal distribution. G: Distribution of the low-band frontal slopes of the continuous data from Dataset 3 for each participant individually. X-axis exhibits low-band frontal slope values.


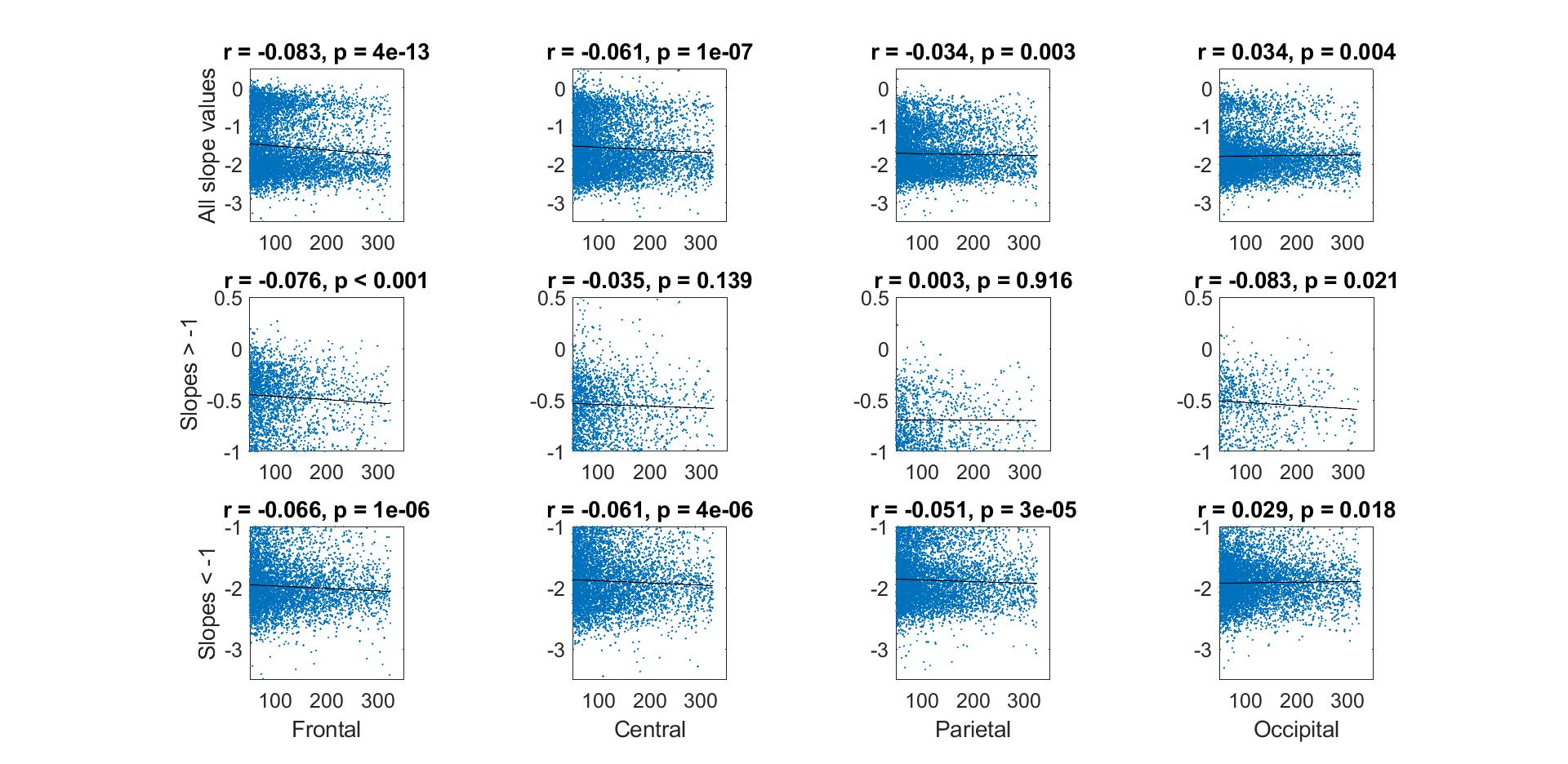


**B: 5 – 30Hz**

**A: 2 – 30Hz**

**C: 5 – 30Hz**

**\**

## **Supplementary Figure 3. Continuous data of Dataset 3: between-episode analysis.**

**A – B: Aperiodic slopes and EM amplitudes.** Correlations between the low-band aperiodic slopes in the 2 – 30Hz (A) and 5 – 30Hz (B) bands for each topographical area and EM amplitudes are shown separately for all slopes (first row, bimodal distribution), slopes > -1 (values more typical for the wake, second row, almost unimodal distribution) and slopes < -1 (values more typical for sleep, third row, almost unimodal distribution). Each dot represents a four-second epoch from the continuous data of Dataset 3 (20 participants, 7554 epochs), x-axis exhibits the EM amplitudes in μV, r – Spearman’s correlation coefficient, EM – eye movements. **C: Frequency distribution of the slopes in the 5 – 30 Hz band.** Aperiodic slopes calculated from the continuous data of Dataset 3 over frontal, central, parietal and occipital electrodes separately show bimodal distribution over the frontal area.

## **Electrocardiography (ECG)**

Given that recently, it has been shown that “cortically” measured aperiodic activity could be (at least partly) attributed to cardiac activity captured by surface electrodes via volume conduction (Schmidt et al., 2023), we also analyzed aperiodic activity over the ECG channel. The procedure was the same as for the EEG channels described in Methods. The ECG signal was recorded as part of the EEG recordings and was available for Datasets 1 and 2.

We found comparable cardiac low-band (2 – 30Hz) aperiodic slopes during tonic and phasic states (Study 1: -1.84 ± 0.30 vs -1.83 ± 0.32; Study 2: -1.99 ± 0.44 vs -2.01 ± 0.46). In addition, to confirm that the aperiodic ECG signal relates to heart rate we calculated the mean heart rate (i.e., 60 s/R-R interval s). The R peaks were detected by the *findpeaks* function and checked through the visual inspection for each 4s epoch. Then, we correlated the aperiodic cardiac slopes with heart rate averaged over each epoch using Pearson’s correlations within each participant individually. Significant correlations were observed in about 37.5% of all participants only.
